# Non-small cell lung cancer is characterised by a distinct inflammatory signature in serum compared with chronic obstructive pulmonary disease

**DOI:** 10.1038/cti.2016.65

**Published:** 2016-11-02

**Authors:** Hanne Astrid Eide, Ann Rita Halvorsen, Vandana Sandhu, Anne Fåne, Janna Berg, Vilde Drageset Haakensen, Elin H Kure, Odd Terje Brustugun, Cecilie Essholt Kiserud, Jon Amund Kyte, Åslaug Helland

**Affiliations:** 1 Department of Cancer Genetics, Institute for Cancer Research, Oslo University Hospital-The Norwegian Radium Hospital, Oslo, Norway; 2 Department for Cell Therapy, Oslo University Hospital-The Norwegian Radium Hospital, Oslo, Norway; 3 Department of Medicine, Vestfold Hospital Trust, Tønsberg, Norway; 4 Department for Environmental Health and Science, Telemark University College, Bø in Telemark, Norway; 5 Department of Oncology, Oslo University Hospital-The Norwegian Radium Hospital, Oslo, Norway; 6 Department of Oncology, National Advisory Unit on Late Effects After Cancer Treatment, Oslo University Hospital-The Norwegian Radium Hospital, Oslo, Norway

## Abstract

Development of lung cancer is closely related to smoking in a majority of patients. Most smokers, however, do not develop lung cancer in spite of a high mutational load accumulating in the lung tissue. Here we investigate whether a cancer-specific footprint can be revealed by investigating circulating inflammatory markers in patients with non-small cell lung cancer (NSCLC) compared with patients with chronic obstructive pulmonary disease (COPD), both cohorts characterised by similar smoking history. Serum concentrations of 57 cytokines and matrix metalloproteinases (MMPs) from 43 patients with advanced NSCLC were evaluated by multiplex immunoassays and compared with serum samples from 35 patients with COPD. Unsupervised hierarchical clustering and non-parametric analyses were performed. False discovery rate was used to adjust for multiple testing. Clustering of cytokine and MMP concentrations in the serum revealed a distinct separation of the NSCLC patients from the COPD group. Individual concentrations of thymus and activation-regulated cytokine (C-C motif chemokine ligand 17), Gro-b (C-X-C motif chemokine ligand 2 (CXCL2)), CXCL13, interleukin (IL)-1ra, IL-6, IL-8 (CXCL8), IL-16, IL-17A, macrophage migration inhibitory factor (MIF), granulocyte colony-stimulating factor, platelet-derived growth factor subunit B, MMP-2, MMP-8 and MMP-12 were significantly different in serum from NSCLC and COPD patients. Moreover, the interferon-γ/IL-10 ratio was lower in cancer patients compared with COPD patients, consistent with a cytokine milieu favouring tumour tolerance. Our results suggest that NSCLC is characterised by a distinct inflammatory signature in serum. The different cytokine profiles in NSCLC and COPD patients may represent tumour-promoting and tumour-suppressing immune responses developing in response to mucosal inflammation and mutations induced by smoking.

The severity of lung cancer is well known and lung cancer remains the leading cause of cancer-related death worldwide.^1^ Treatment and prognosis rely heavily on disease stage at diagnosis. A majority of patients are diagnosed in advanced stages, not eligible for treatment with curative intent. Although scientific endeavours are comprehensive, there is a definite need for further insight into lung cancer biology aiming at improved diagnostics, more efficient cancer treatment and ultimately an increase in survival.

Inflammation is linked to multiple tumour-promoting effects and is in general recognised to have an important part in cancer evolution.^2^ The lungs are vulnerable for air-borne environmental factors, and tobacco smoke in particular is implicated in lung inflammation.^3, 4^ An association between the inflammatory disease chronic obstructive pulmonary disease (COPD) and lung cancer is evident from epidemiological and clinical studies.^5, 6^ There is, however, limited knowledge if or how the host immune response influence whether an individual with COPD develops lung cancer.

We know that heavy smokers accumulate a high mutational load in the lung tissue, but still only a minority develop cancer. Carcinogenesis is not merely a malignant transformation of cells; it is dependent on multiple interactions with different cell types making up the tumour microenvironment. Cells of the immune system, endothelial cells and fibroblasts are among others found adjacent to malignant cells in a tumour and are of importance in cancer development and progression.^7^ Genetic events in cells are followed by an interaction with the immune system.^2^ The immune response can either suppress the development of a tumour by eliminating cancer cells or promote tumour growth by selecting cancer cells that escape the immune control.^8^ It is evident that immune cells in the tumour microenvironment are relevant for tumour characteristics and patient outcome. Tumour-infiltrating lymphocytes are of clinical impact in several cancers, including lung cancer where high levels are correlated to better prognosis.^9^ To target the non-malignant cells in the tumour environment is an interesting approach to the treatment of cancer. Certainly, recent clinical trials with immune-associated mediators have proven efficient in non-small cell lung cancer (NSCLC) treatment.^10, 11^

Proteins and enzymes make up complex networks responsible for cell-to-cell communication in the tumour microenvironment. Cytokines are essential modulators. A shift from an immunological pattern with a T helper type 1 (T_H_1) orientation to a T_H_2 pattern mediated by cytokines is reported as a biological event in the carcinogenesis.^12^ Indeed, increased serum levels of specific cytokines are associated with a risk of developing lung cancer and is linked to survival both in early- and advanced-stage lung cancer.^13, 14, 15^ Matrix metalloproteinases (MMPs) are proteolytic enzymes originally known to degrade extracellular matrix. Moreover, they also regulate the activity of growth factors, cytokines, cell receptors and other proteases, thus influencing a variety of inflammatory processes and biological activities in cancer.^16^

Here we analyse a comprehensive panel of circulating cytokines and MMPs in patients with advanced-stage NSCLC using COPD patients as a control group. Both cohorts represent current or former smokers. Our aim is to investigate the cancer-specific footprint in the serum mirroring lung cancer carcinogenesis.

## Results

### Patient and disease characteristics

The median age of the NSCLC patients at inclusion was 70 years (range 47–88) and 72% of the patients were male. All NSCLC patients had a history of smoking either as current (26%) or former (74%) smokers. Twenty six of the patients (61%) had not received previous chemotherapy. Fourteen NSCLC patients (33%) were treated with systemic steroids at the time of inclusion. The median age of the COPD patients were 72 years (range 50–87) and 40% were male. All but three patients (91%) had a smoking history. Only 4 patients (11%) in the COPD group received systemic steroids at the time of serum sampling. A majority had grade III COPD disease (46%). The NSCLC and COPD cohorts were fairly well balanced at baseline with no significant differences in age and smoking history. A higher percentage of patients in the NSCLC cohort (33%) used systemic steroids compared with the COPD cohort (11%), but this difference was not significant. There was, however, a significant difference in sex (*P*=0.006) with a larger percentage of males relative to females in the NSCLC group compared with the COPD group. Baseline characteristics of the NSCLC and COPD patients are shown in Table 1.

### Distinct signatures of serum proteins separating NSCLC from COPD patients

**Figure 1:**
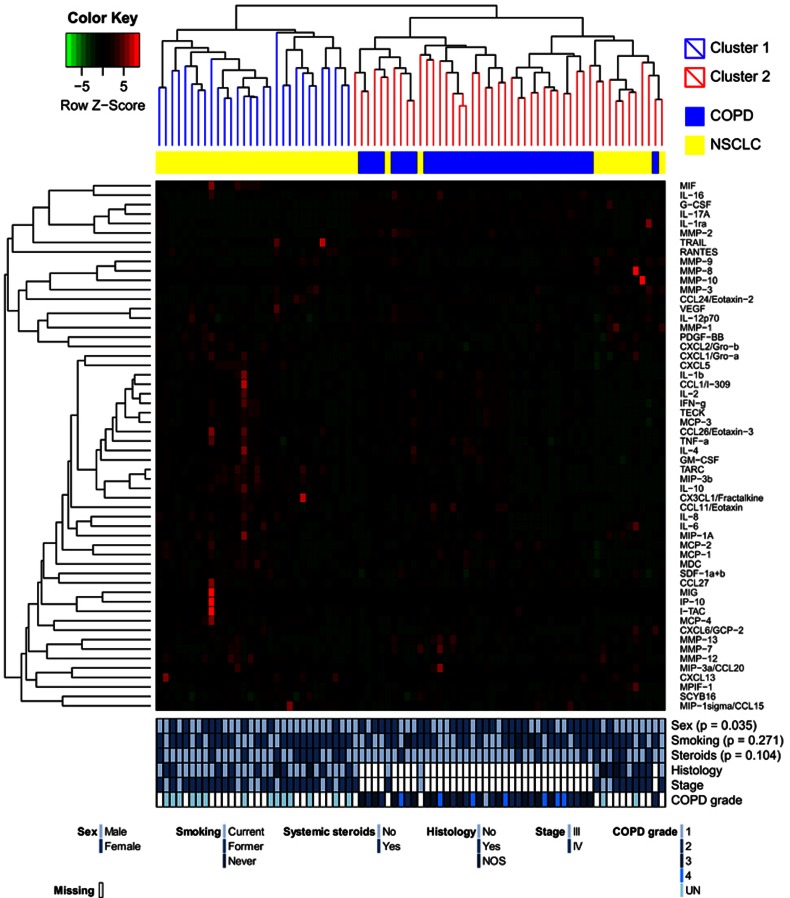
Hierarchical clustering of 57 proteins (cytokines and MMPs) in serum samples from 43 patients with NSCLC and 35 patients with COPD. Clinical parameters with tests for significant differences between the clusters are visualised below the heat map. AD, adenocarcinoma; NOS, none otherwise specified; SCC, squamous cell carcinoma; UN, unknown COPD grade.

Hierarchical clustering of cytokines and MMPs separated the serum samples into two clusters (Figure 1) illustrating a clear separation of cytokine levels in NSCLC and COPD patients. There was a significant difference between the two clusters regarding sex (*P*=0.035), concurrent with the difference in baseline characteristics with fewer men among the COPD patients. No significant differences in smoking history or in the use of systemic steroids were seen between the clusters.

**Figure 2:**
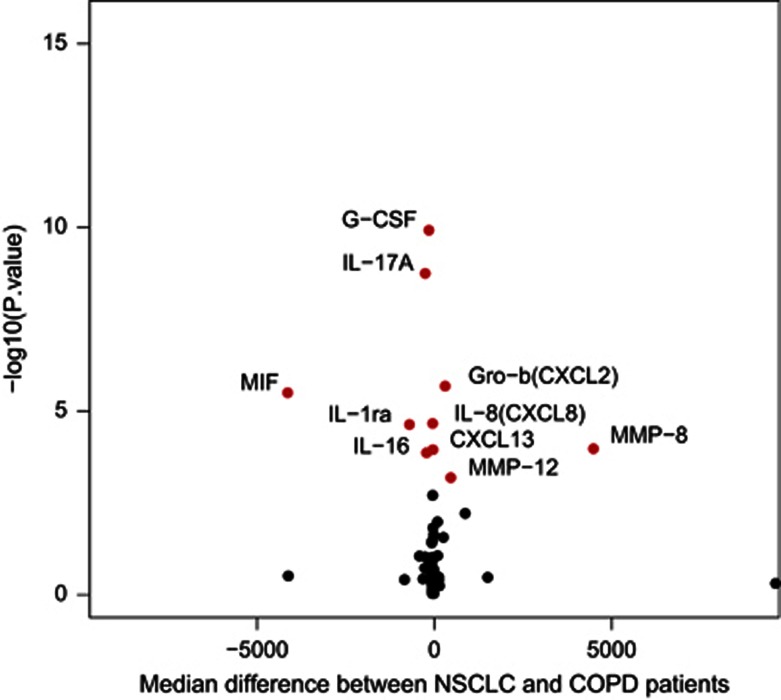
Volcano plot illustrating the magnitude and significance of the differences in cytokine/MMP serum concentration levels in patients with NSCLC and COPD. Dots marked in red are cytokines/MMPs with a significantly different median serum concentration after correction for multiple testing.

Several individual cytokines and MMPs had different concentrations in serum between the two cohorts (Table 2, Figure 2). After correction for multiple testing, NSCLC patients had a significant higher median level of thymus and activation regulated cytokine (TARC; C-C motif chemokine ligand 17 (CCL17)), Gro-b (C-X-C motif chemokine ligand 2 (CXCL2)), CXCL13, interleukin (IL)-6, IL-8 (CXCL8), platelet-derived growth factor subunit B (PDGF-BB), MMP-8 and MMP-12 in the serum compared with the COPD controls. For IL-1ra, IL-16, IL-17A, macrophage migration inhibitory factor (MIF), granulocyte colony-stimulating factor (G-CSF) and MMP-2, the median serum concentration value in NSCLC patients was significantly lower compared with COPD patients. Box plots of significantly different levels of cytokines/MMPs, and a complete table of all proteins investigated in the two cohorts are included in Supplementary Information (Supplementary Figure S1 and Supplementary Table S1).

Interferon gamma (IFNγ) and IL-10 are considered hallmark cytokines for antitumour T_H_1 responses and immunological tumour tolerance, respectively. The serum concentration of IFNγ did not vary between NSCLC and COPD patients. The IL-10 levels were higher in NSCLC patients but did not retain significance after correction for multiple testing. IFNγ/IL-10 ratio, however, was significantly different between NSCLC and COPD patients (*P<*0.001), with a median ratio of 0.84 in the NSCLC group versus 1.11 in the COPD cohort (Figure 2).

**Figure 3:**
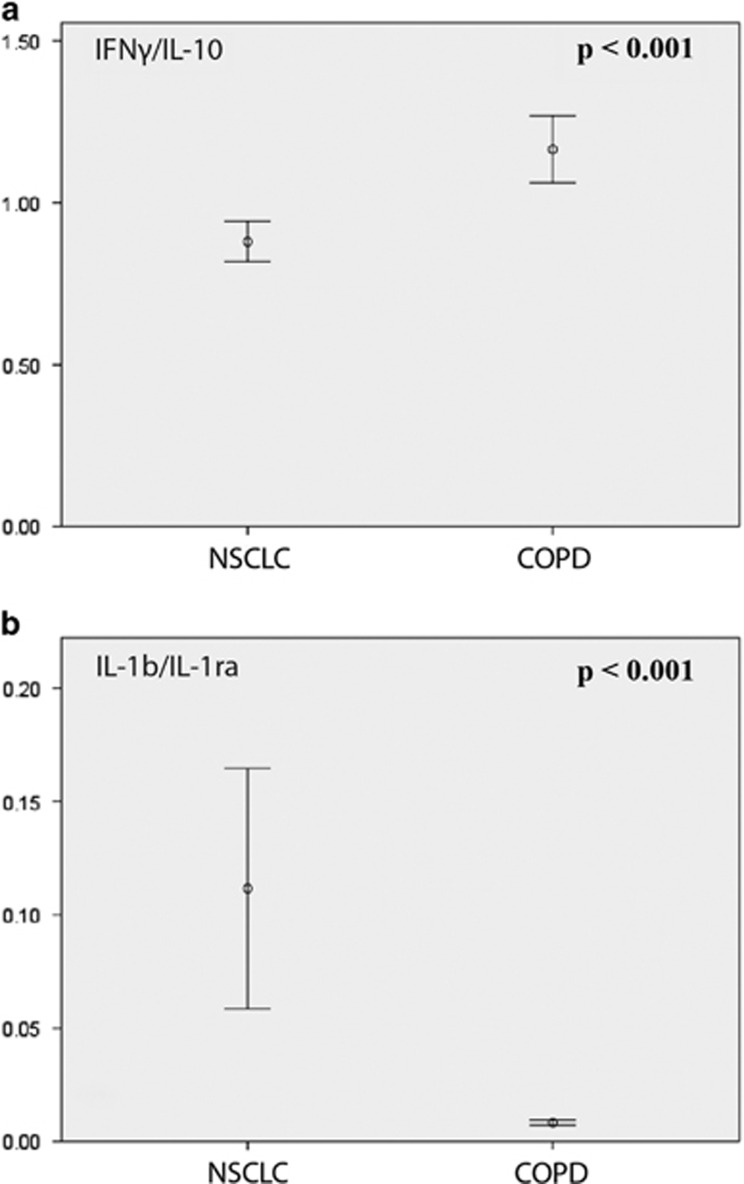
Differences in IFNγ/IL-10 (**a**) and IL-1b/IL-1ra (**b**) ratios comparing patients with NSCLC and COPD.

Another interesting connection at the biological level in this panel of cytokines was the association between the pro-inflammatory protein IL-1b and its naturally occurring antagonist IL-1ra. No difference was seen in the level of IL-1b between the groups, but the IL-1ra concentration in the serum samples was significantly lower in the NSCLC group. Accordingly, the IL-1b/IL-1ra ratio in the NSCLC group was significantly higher compared with the COPD patients (*P<*0.001; Figure 3).

### Associations of cytokine and MMP levels in different clinical subsets of NSCLC patients

When omitting all NSCLC and COPD patients using corticosteroids, we observed a distinct serum signature separating NSCLC and COPD patients, similar to the one observed in the entire cohort (Supplementary Table S2). In addition, the levels of IL-10 and MMP-3 were observed to be significantly different, with a higher and lower median level in NSCLC patients compared with COPD patients, respectively. The significant association of TARC between the patient groups was lost.

Among NSCLC patients, two MMPs had a significant different concentration after correction for multiple testing, when comparing patients using systemic steroids or not (Table 3). MMP-3 (*P<*0.001) and MMP-9 (*P*=0.001) had a higher median concentration in the systemic steroid-user group.

Cytokine/MMP levels in other clinical subsets of NSCLC are presented in Supplementary Table S3. Of note, current smokers had a significantly higher level of cytokines CCL11 (*P*=0.001), Gro-a (CXCL1) (*P*=0.001), stromal-derived factor 1 alpha+beta (SDF-1a+b; CXCL12) (*P*=0.003) and IL-4 (*P<*0.001) compared with former smokers. Increased C-reactive protein (CRP) levels correlated with higher levels of multiple pro-inflammatory cytokines. The association between CRP, Gro-b (CXCL2) and IL-6 remained statistically significant after correction for multiple testing (*P*=0.001 and *P<*0.001, respectively). No significant differences were seen after correction for false discovery rate in NSCLC patients with a previous history of chemotherapy use compared with patients with no prior chemotherapy treatment.

## Discussion

In the present study, we have investigated a panel of 57 circulating inflammatory markers consisting of cytokines and MMPs in the serum from NSCLC patients with advanced disease. The cytokines and MMPs were chosen based on previously published reports on potential biomarkers in lung cancer, and they represent a broad spectrum of inflammatory mediators of special interest in the tumour microenvironment. Obtaining peripheral blood samples is easy compared with samples of lung tumours. The concept of revealing essential markers of disease in blood tests have a particular allure in this cohort of advanced-stage NSCLC patients, where life expectancy is short and good quality of life is important.

To the best of our knowledge, this is the first study where COPD patients are chosen as controls in a serum cytokine/MMP analysis in NSCLC patients. Several cytokine/MMP studies have previously been conducted with healthy subjects as controls.^15, 17^ All of the NSCLC patients included in our study were either former or current smokers, and there were no significant differences in pack years between the NSCLC and COPD cohorts. COPD is an inflammatory disease strongly associated with smoking and is therefore likely to have an effect on the cytokine profile in patients. Higher levels of inflammatory markers have been reported among former and current smokers as well as among those with a history of chronic bronchitis or emphysema, compared with healthy subjects, supporting that notion.^13^ Respiratory function tests were not a requirement at inclusion in the Thoracal Radiotherapy and Tarceva (ThoRaT) study. Sixteen of the NSCLC patients reported to have COPD (degree unknown) in their medical history at baseline. Owing to the similar smoking histories in both cohorts, it is likely that even more of the NSCLC patients would be suffering from COPD. COPD patients, on the other hand, were followed for a minimum of 2 years after serum sampling, and none of the patients included in theseanalyses developed cancer later. In our study, we aimed at discovering a cytokine and MMP profile in the serum reflecting the malignant disease and not a confounding inflammatory pattern owing to smoking. The different cohorts have a similar background, the use of a highly carcinogenic substance, but only one group of patients developed cancer.

The study investigates serum cytokine profiles in a relatively small cohort of patients with advanced NSCLC. Previous chemotherapy could be a possible confounder in the evaluation of the differences in cytokine distribution between NSCLC and certainly untreated COPD patients. Sixty percent of the NSCLC patients included in the study, however, had not received treatment with chemotherapy prior to inclusion. Moreover, when comparing the cytokine concentration level in NSCLC with and without previous chemotherapy, no significant differences were found.

Many former studies have been conducted with only a few cytokines in parallel in the same individuals, but some have reported on larger panels. Kaminska *et al.*^17^ found elevated serum levels of tumour necrosis factor alpha (TNFα), IL-6, IL-8, IL-10, IL-1ra, vascular endothelial growth factor (VEGF), granulocyte-macrophage colony-stimulating factor (GM-CSF) and G-CSF in a study including NSCLC patients of all stages, compared with healthy controls. Elevated levels of IL-6, IL-8 and IL-10 were consistent with our findings, but contrary to their study, we found lower levels of G-CSF and IL-1ra, and no differences in TNFα, VEGF and GM-CSF in comparison with COPD patients. In another study, Barrera *et al.*^15^ found higher levels of IL-6, IL-8, IL-12p70, IL-17A and IFNγ comparing an advanced NSCLC cohort with 50% non-smokers, with healthy controls. Again, our results confirm the high levels of IL-6 and IL-8 in NSCLC patients; however, no differences were seen between our cohorts in IL-12p70 or IFNγ concentrations, and IL-17A was found to be lower in our NSCLC group. COPD-associated inflammation can lead to elevated serum levels of multiple cytokines and the different observations with regard to some cytokines between our study and those of Karminska *et al.*^17^ and Barrera *et al.*^15^ may be explained by our use of COPD controls, rather than healthy individuals.

The concentration of IL-17A in serum was found approximately fourfold lower in NSCLC versus COPD patients in our study. We cannot, from our data set, decode if this finding reflects elevated levels of IL-17A in the COPD cohort or low levels among the NSCLC patients. Based on current knowledge, the first explanation appears more likely, as IL-17 has been found elevated in patients with a range of chronic inflammatory disorders.^18^ One former study revealed elevated levels of IL-17A in serum collected from COPD patients compared with control groups of both healthy smokers and non-smokers; IL-17A was also seen increased in accordance with advancing COPD stages.^19^ Studies in NSCLC patients have, contradictory to our results, found elevated IL-17A levels in circulation but then again compared with healthy controls.^15, 20^ It is known that naive CD4+ T_H_ precursor cells can differentiate into a variety of different T_H_ subsets, including T_H_1, T_H_2, T_H_17 and regulatory T cells.^21^ T_H_17 cells are considered the predominant producer of IL-17. Both tumour-suppressing and tumour-promoting functions have been attributed to IL-17A and T_H_17 cells, and the role of T_H_17 cells in tumour immunity remains ambiguous.^22^

The immune system has a pivotal role against cancer. The development of a successful immune response depends on the balance between the T_H_1 (antitumour) and T_H_2 responses and on the activity of immune cells that inhibit antitumour responses. T cells producing IL-10, in particular type 1 regulatory (Tr1) T cells and T_H_2 cells, are considered to be key suppressors of antitumour response and it is previously proposed that T_H_2-type inflammation facilitates tumour growth.^23^ It is further known that T_H_1 cells produce one particular set of cytokines such as IL-2, IFNγ and TNFα while T_H_2 cells produce others, for example, IL-4, IL-5, IL-6, IL-10 and IL-13.^24^ In our study, IL-6 and IL-10 concentration in the serum was found to be higher in NSCLC patients, although the differences in IL-10 did not retain significance after correction for multiple testing. IFNγ/IL-10 ratio was nonetheless found reduced in NSCLC patients compared with the COPD group, supporting the notion that the T_H_1 response in NSCLC patients was turned down, whereas the T_H_2 response was more prominent. These contrasting immune responses developing in different individuals may carry particular importance for the development of lung cancer. Intriguingly, most heavy smokers do not develop lung cancer in spite of a high mutation load accumulating in the lung tissue. There is increasing evidence suggesting that this resilience is related to the host immune response, known to be particularly potent for controlling tumours with a high mutation load and thus a high neoantigen frequency.^25, 26, 27^ We hypothesise that the different cytokine profiles identified among NSCLC patients and COPD subjects in our study represent a footprint of tumour-promoting versus tumour-suppressing immune responses developing in the host, in response to mucosal inflammation and mutations induced by smoking. It would be of interest to assess how this cytokine profile is influenced by immunological checkpoint inhibitors or other forms of therapy. Further, it is possible that a pro-cancer cytokine profile precedes the clinical manifestation of NSCLC and can be used for early detection of lung cancer.

The pro-inflammatory cytokine IL-1 has been shown elevated in several cancer types such as breast, colon, melanoma, head and neck as well as lung cancer and is associated with an increase in metastases and poor prognosis.^28^ We did not observe any difference between serum levels of IL-1b between the NSCLC and COPD cohorts investigated. The NSCLC patients in our study did, however, have a median concentration level of IL-1ra approximately half the value seen in the COPD serum samples. Moreover, the median IL-1ra concentration values were lower in stage IV NSCLC patients compared with patients in stage III, although this did not retain significance after correction for multiple testing. IL-1ra is a naturally occurring IL1 antagonist. In IL-1ra-deficient mice, skin tumours developed more rapidly compared with wild-type mice in a model of IL-1-induced carcinogenesis.^29^ In lung cancer, elevated levels of IL-1ra in serum have previously been found associated with decreased risk of lung cancer.^13^ Here we discovered that the IL-1b/IL-1ra ratio was elevated in NSCLC patients compared with COPD patients, indicating that the pro-malignant IL-1b expression level was higher in NSCLC patients relative to the antagonist; the ‘cancer-protective' IL-1ra. Interestingly, therapeutic agents reducing IL-1 activity, such as recombinant IL-1ra, are commercially available for treatment of inflammatory diseases and could be an approach in cancer treatment.^30^

Several meta-analyses suggest MMPs as prognostic markers in lung cancer, although the results are conflicting.^31, 32, 33^ Many previous studies of MMPs comparing lung cancer patients with healthy individuals have also shown contradictory results.^34^ In our study, a higher level of circulating MMP-8 and MMP-12 was revealed, whereas MMP-2 was reduced in NSCLC compared with COPD patients. When excluding patients using steroids from both cohorts, MMP-3 was also found lower in NSCLC patients. In the NSCLC cohort, MMP-3 and MMP-9 levels were elevated in patients using systemic steroids. It is previously shown that MMP-3 can be induced by steroids.^35^ MMP-9 may also be released from an increasing amount of neutrophils accompanying treatment with steroids. In COPD, tissue destruction and remodeling are important processes where MMPs are likely to have a central part.^36^ The most likely explanation for MMPs in the pathogenesis of cancer is also through degradation of extracellular matrix enabling tumour cell invasion and metastasis. However, MMPs are also recognised as important participants in the communication between cancer cells and the non-malignant stroma and can modulate most stages of tumour progression.^16^

Our study suggests that NSCLC is mirrored by a distinct inflammatory signature in the serum. The different cytokine profiles in NSCLC and COPD patients may represent tumour-promoting and tumour-suppressing immune responses. Lack of independent validation remains a limitation in the interpretation of the data as well as the relatively small number of patients. However, the results are encouraging for a follow-up in a larger cohort owing to its possible implications for disease etiology, diagnostics and treatment. There is an increasing interest in the use of radiological screening for early detection of lung cancer. In this setting, serum biomarkers may serve as a cost-effective, high-throughput tool for identifying subjects at risk who may be referred to computed tomographic scans.

## Methods

### Study population and data collection

The cytokine analyses were performed using serum samples obtained from 43 patients with advanced-stage NSCLC included in an ongoing clinical study (ThoRaT) from December 2011 until June 2015. The patients were referred for palliative radiotherapy. Clinical characteristics of the NSCLC patients were collected from the hospital medical records. Tumours were staged according to the Union for International Cancer Control, Tumour, Node, Metastasis 7. Histopathological evaluations were retrieved from pathology reports.

### Blood sample processing

The blood samples from the NSCLC patients were collected at inclusion in the ThoRaT study, prior to radiotherapy. Blood was collected in serum tubes, kept in room temperature appending coagulation and then processed at 2450 *g* for 15 min within 1 h after sampling. Finally, the samples were transferred in 250 μl aliquots into cryovials and stored at −80 °C until usage.

### COPD cohort

Serum samples from 35 patients with COPD were obtained at the Department of Medicine, Vestfold Hospital Trust, Tønsberg, Norway. Clinical information was acquired from the hospital records (Table 1). All COPD patients included were in a regular follow-up and had no sign of lung cancer prior to blood sampling. The patients were also followed for a minimum of 2 years after blood sampling with no sign of cancer. The serum samples in the COPD cohort were preprocessed under strictly defined and equal conditions as the samples obtained from the NSCLC patients, stored at the same site as the NSCLC samples and processed further at the same centre by the same personnel.

### Cytokine analyses

Serum concentration levels of the cytokines CCL1/I-309, monocyte chemotactic chemokine-1 (MCP-1) (CCL2), MCP-2 (CCL8), MCP-3 (CCL7), MCP-4 (CCL13), RANTES (CCL5), macrophage inflammatory protein (MIP) 1alpha (CCL3), MIP-1sigma (CCL15), MIP-3alpha (CCL20), MIP-3beta (CCL19), TARC (CCL17), eotaxin (CCL11), eotaxin-2 (CCL24), eotaxin-3 (CCL26), CCL-21, macrophage-derived chemokine (CCL22), myeloid progenitor inhibitory factor 1 (CCL23), thymus expressed cytokine (CCL25), CCL27, growth-regulated protein alpha (Gro-a) (CXCL1), growth-regulated protein beta (Gro-a) (CXCL2), CXCL5, granulocyte chemotactic protein-2 (CXCL6), monokine induced by gamma interferon (CXCL9), interferon gamma induced protein-10 (CXCL10), interferon inducible T-cell alpha chemoattractant (CXCL11), SDF-1a+b (CXCL12), CXCL13, SCYB16 (CXCL16), fractalkine (CX3CL1), IL-1b, IL-1ra, IL-2, IL-4, IL-6, IL-8, IL-10, IL12p70, IL-16, IL17a, IFNγ, TNFα, TNF-related apoptosis-induced ligand, MIF, G-CSF, GM-CSF, PDGF-BB, VEGF and MMP-1, -2, -3, -7, -8, -9, -12 and -13 were quantified using a multiplex bioassay (BioRad, Hercules, CA, USA) according to the manufacturer's instructions. Concentrations were calculated using an eight-parameter standard curve. Each serum sample was run in duplicates and averaged to calculate the concentrations.

### Statistical analyses

Data are reported using descriptive statistics with percentages, means, medians and ranges. Unsupervised hierarchical clustering was performed using Spearman correlation and average linkage with scaled cytokine/MMP values owing to differences in interprotein concentration values. Differences in the clinical groups and between clusters were calculated with two-sided *t*-tests (assuming equal variance) and chi-squared/Fisher's exact tests for continuous and categorical data, respectively. Non-parametric tests, Mann–Whitney *U*-test and Kruskal–Wallis test, were used to explore the differences in individual cytokine levels between NSCLC and COPD patients as well as in different clinical subsets. The median difference between serum levels in the two cohorts was utilised to make the volcano plot, also owing to lack of normally distributed data. The Benjamini–Hochberg false discovery rate was used in order to reduce the possibility for significant independent test results by chance alone owing to multiple testing.

Data were analysed using the SPSS software package version 21 (SPSS, Chicago, IL, USA). Hierarchical clustering performed in R version 3.2.2 (R Project for Statistical Computing, Vienna, Austria). Two sided *P*-values<0.05 were considered statistical significant. Stricter levels of significance due to correction for multiple testing are noted in the text and tables where applicable.

### Ethics approval

This study was approved by the regional ethics committee in Norway (reference number 2012/320) and the institutional review board. A written consent was obtained from all the participants in this study.

## Figures and Tables

**Table 1:** Characteristics of NSCLC and COPD patients included in the cytokine/MMP analysis

*Variable*	*NSCLC cohort*		*COPD cohort*		*P-value*
	N=*43*	*%*	N=*35*	*%*	
*Age at inclusion (years)*					
Mean/median/range	69/70/47–88		71/72/50–87		0.333^a^
					
*Sex*					
Male	31	72.1	14	40.0	0.006^b^
Female	12	27.9	21	60.0	
					
*Smoking history*					
Current	11	25.6	13	37.1	0.057^c^
Former	32	74.4	19	54.3	
Never	0	0	3	8.6	
					
*Pack years*					
Mean/median/range	34/30/4–144		32/35/12–60		0.698^a^
					
*Use of systemic steroids*					
Yes	14	32.6	4	11.4	0.153^b^
No	29	67.4	31	88.6	
					
*COPD grade*					
I			1	2.9	
II			10	28.6	
III			16	45.7	
IV			8	22.8	
					
*ECOG performance status*					
0	8	18.6			
1	22	51.2			
2	13	30.2			
					
*Stage*					
III	11	25.6			
IV	32	74.4			
					
*Histology*					
Adenocarcinoma	26	60.5			
Squamous cell carcinoma	13	30.2			
NOS	4	9.3			
					
*Previous chemotherapy*					
Yes	17	39.5			
No	26	60.5			

Abbreviations: COPD, chronic obstructive pulmonary disease; ECOG, Eastern Cooperative Oncology Group; MMP, matrix metalloproteinase; NOS, not otherwise specified; NSCLC, non-small cell lung cancer.

a Two sided *t*-test for continuous variables.

b Fisher's exact test for categorical variables.

c Chi-square test for categorical variables.

**Table 2:** Levels of circulating cytokines and MMPs with a significant difference in NSCLC and COPD patients

*Cytokine/MMP*	*NSCLC median*	*COPD median*	*P-value*
CCL1/I-309	77	89	0.041
MIP-3a (CCL20)	24	17	0.036
MIP-3b (CCL19)	919	606	0.029
TARC (CCL17)	400	253	0.011^a^
Eotaxin (CCL11)	63	72	0.037
Gro-b (CXCL2)	878	514	<0.001^a^
CXCL13	54	36	0.000^a^
Fractalkine (CX3CL1)	254	212	0.025
IL-1ra	434	1079	<0.001^a^
IL-6	27	16	0.002^a^
IL-8 (CXCL8)	31	21	0.000^a^
IL-10	76	56	0.016
IL-16	370	532	<0.001^a^
IL-17A	73	273	<0.001^a^
MIF	808	4895	<0.001^a^
G-CSF	33	127	<0.001^a^
PDGF-BB	3288	2357	0.003^a^
MMP-2	37 242	89 243	<0.001^a^
MMP-8	9436	5072	<0.001^a^
MMP-12	871	347	<0.001^a^

Abbreviations: CCL, C-C motif chemokine ligand; COPD, chronic obstructive pulmonary disease; CXCL, C-X-C motif chemokine ligand; G-CSF, granulocyte colony-stimulating factor; IL, interleukin; MIF, macrophage migration inhibitory factor; MIP, macrophage inflammatory protein; MMP, matrix metalloproteinase; NSCLC, non small cell lung cancer; PDGF-BB, platelet-derived growth factor subunit B; TARC, thymus and activation regulated cytokine. Concentrations measured in pg ml^−1^.

a Statistically significant *P*-values retained after correction for multiple.

**Table 3:** Cytokine and MMP serum concentration values in the NSCLC patients (*N*=43) with a significant different distribution in non-steroid users compared with patients using systemic steroids

*Cytokine/MMP*	*NSCLC cohort*		*P-value*
	*No systemic steroids,* N=*29, median*	*Systemic steroids,* N=*14, median*	
MDC (CCL22)	977	453	0.013
IL-17a	88	56	0.011
MMP-3	5681	15 443	<0.001^a^
MMP-7	3759	2676	0.036
MMP-9	42 768	77 164	0.001^a^
MMP-12	1050	389	0.003
MMP-13	148	75	0.011

Abbreviations: CCL, C-C motif chemokine ligand; IL, interleukin; MDC, macrophage-derived chemokine; MMP, matrix metalloproteinase; NSCLC, non-small cell lung cancer.

Concentrations measured in pg ml^−1^.

a Statistically significant *P*-values adjusted for multiple testing.
