# Supplementary material for: Non-small cell lung cancer is characterised by a distinct inflammatory signature in serum compared with chronic obstructive pulmonary disease
Source: Clin Transl Immunology. 2016 Nov 2;5(11):e109–. doi: 10.1038/cti.2016.65 (PMC5133367; doi:10.1038/cti.2016.65)
Supplement: Supplementary Information [file cti201665x1.pdf]

Supplementary information

Table S1. Circulating cytokines and MMPs in NSCLC and COPD patients with median concentrations values and ranges.

**Cytokine**	**NSCLC subjects Median (range)**	**COPD subjects Median (range)**	**p-value**
CCL1/I-309	77 (48-283)	89 (65-128)	**0.041**
MCP-1 (CCL2)	74 (11-203)	80 (32-172)	0.550
MCP-2 (CCL8)	61 (13-192)	80 (25-164)	0.190
MCP-3 (CCL7)	87 (35-153)	96 (57-150)	0.133
MCP-4 (CCL13)	81 (32-358)	80 (47-173)	0.662
RANTES (CCL5)	11872 (6271-25588)	15941 (6119-22197)	0.327
MIP-1A (CCL13)	15 (8-49)	16 (10-27)	0.615
MIP-1sigma (CCL15)	9424 (675-20703)	7859 (3778-44283)	0.358
MIP-3a (CCL20)	24 (10-195)	17 (9-303)	**0.036**
MIP-3b (CCL19)	919 (130-2777)	606 (213-1720)	**0.029**
TARC (CCL17)	400 (49-1561)	253 (81-977)	**0.011***
Eotaxin (CCL11)	63 (31-128)	72 (45-178)	**0.037**
Eotaxin-2 (CCL24)	383 (74-1846)	484 (66-1054)	0.402
Eotaxin-3 (CCL26)	52 (14-227)	48 (26-85)	0.530
CCL21	6399 (3636-8571)	6607 (2434-11019)	0.098
MDC (CCL22)	795 (179-2566)	1006 (310-1836)	0.200
MPIF-1 (CCL23)	473 (32-1875)	427(135-915)	0.222
TECK (CCL25)	707 (363-1437)	787 (457-1192)	0.131
CCL27	1635 (716-5984)	1450 (446-2748)	0.436
Gro-a (CXCL1)	320 (152-623)	299 (215-437)	0.557
Gro-b (CXCL2)	878 (155-1641)	514 (71-797)	**<0.001***
CXCL5	875 (121-2221)	807 (219-1439)	0.537
GCP-2 (CXCL6)	45 (10-118)	34 (22-97)	0.197
MIG	749 (114-24748)	599 (222-1458)	0.092
I-TAC	44 (10-417)	38 (13-138)	0.100
IP-10	230 (66-11983)	223 (64-524)	0.424
SDF-1a+b	2317 (746-4158)	2117 (658-3424)	0.612
CXCL13	54 (27-842)	36 (15-334)	**<0.001***
SCYB16	562 (116-954)	591 (206-862)	0.313
Fractalkine (CX3CL1)	254 (96-1250)	212 (120-491)	**0.025**
IL-1b	8.5 (2.3-34.3)	7.6 (4.8-19.9)	0.124
IL-1ra	434 (14-4161)	1079 (748-1396)	**<0.001***
IL-2	21 (7-77)	23 (14-53)	0.273
IL-4	24 (13-91)	25 (15-64)	0.988
IL-6	27 (6-103)	16 (10-35)	**0.002***
IL-8 (CXCL8)	31 (9-69)	21 (12-67)	**<0.001***
IL-10	76 (28-198)	56 (18-96)	**0.016**
IL-12p70	70 (6-226)	74 (21-155)	0.517
IL-16	370 (227-1297)	532 (310-1261)	**<0.001***
IL-17A	73 (8-367)	273 (181-386)	**<0.001***
IFNγ	62 (22-179)	63 (39-100)	0.767
TNFα	49 (5-147)	51 (8-116)	0.972
TRAIL	62 (16-641)	66 (25-139)	0.196
MIF	808 (315-67945)	4895 (1067-14729)	**<0.001***
G-CSF	33 (11-234)	127 (90-144)	**<0.001***
GM-CSF	37 (1-114)	299 (215-437)	0.396
PDGF-BB	3288 (461-6538)	2357 (992-4362)	**0.003***
VEGF	106 (6-425)	110 (43-316)	0.837
MMP-1	1481 (81-10606)	1434 (343-6200)	0.988

MMP-2	37242 (10902-85148)	89243 (29936-168547)	**<0.001***
MMP-3	6713 (263-42008)	7497 (2665-33029)	0.413
MMP-7	3247 (755-12000)	3608 (2066-13884)	0.094
MMP-8	9436 (1337-121996)	5072 (1253-17289)	**<0.001***
MMP-9	48102 (3826-152502)	38453 (10500-125935)	0.524
MMP-10	3315 (1190-94090)	3130 (1109-5783)	0.353
MMP-12	871 (52-3088)	347 (131-1183)	**<0.001***
MMP-13	124 (30-532)	156 (46-589)	0.109

Concentration values measured in pg/ml. Statistically significant p-values are written in bold letters. *Statistically significant p-values retained after correction for multiple testing. Abbreviations: NSCLC, non small cell lung cancer; COPD, chronic obstructive pulmonary disease.

Table S2. Significant cytokines and MMPs in serum comparing NSCLC and COPD patients, both in whole cohort and in subjects without use of systemic steroids (significant values only).

**Cytokine/MMP**	**All subjects**			**Subjects without systemic steroids**		
	**NSCLC (n = 43) median**	**COPD (n = 35) median**	**p**	**NSCLC (n = 29) median**	**COPD (n = 31) median**	**p**
CCL1/I-309	77	89	0.041	81	89	0.031
MIP-3a (CCL20)	24	17	0.036	25	17	0.021
MIP-3b (CCL19)	919	606	0.029	948	647	0.015
TARC (CCL17)	400	253	0.011*			
Eotaxin (CCL11)	63	72	0.037	63	72	<0.001*
Gro-b (CXCL2)	878	514	0.000*	880	520	<0.001*
CXCL13	54	36	0.002*	51	33	0.029
MIG (CXCL9)				787	556	0.034
I-TAC (CXCL11)				45	38	0.043
Fractalkine (CX3CL1)	254	212	0.025	254	210	<0.001*
IL1ra	434	1079	0.000*	486	1079	0.001*
IL-6	27	16	0.002*	28	17	<0.001*
IL-8 (CXCL8)	31	21	0.000*	31	21	0.003*
IL-10	76	56	0.016	85	57	<0.001*
IL-16	370	532	0.000*	326	525	<0.001*
IL17A	73	273	0.000*	88	265	<0.001*
MIF	808	4895	0.000*	775	4895	<0.001*
G-CSF	33	127	0.000*	39	127	0.007*
PDGF-BB	3288	2357	0.003*	3444	2357	<0.001*
MMP2	37242	89243	0.000*	37242	89242	0.011*
MMP3				5681	7497	0.008*
MMP8	9436	5072	0.000*	8493	5072	<0.001*
MMP12	871	347	0.000*	1050	347	0.031

Concentration values measured in pg/ml. *Statistically significant p-values adjusted for multiple testing. Abbreviations: NSCLC, non-small cell lung cancer; COPD, chronic obstructive pulmonary disease; MMP, matrix metalloproteinase.

Table S3. Significant different cytokines in clinical subsets of NSCLC patients (p-values in bold letters retained statistical significance after correction by FDR).

	All patients (n=43)		Patients without systemic steroids (n=29)	
**Cytokine**	**Median(range)**	**p – value**	**Median(range)**	**p-value**
**Sex:**				
CCL27				
Female:	2030(1172-5984)	0.016		
Male:	1540(716-2618)			
Eotaxin (CCL11)				
Female:	79(39-128)	0.046		
Male:	62(34-121)			
GM-CSF				
Female:	49(19-114)	0.030		
Male:	35(1-84)			
MMP-3				
Female:			3284(263-25143)	0.018
Male:			6053(3070-34964)	
**Age:**				
CXCL13				
≤60:	41(34-109)	0.032	38(34-47)	0.030
>60:	58(27-842)		54(27-842)	
CCL27				
≤60:	1172(804-1703)	0.022		
>60:	1721(716-5984)			
MIG (CXCL9)				0.016
≤60:			406(237-623)	
>60:			892(120-3578)	
MPIF1 (CCL23)				
≤60:	253(158-335)	0.002	313(158-335)	0.043
>60:	499(32-1875)		505(32-1875)	
**Smoking:**				
CXCL5				
Current:	1089(523-2221)	0.032		
Former:	738(121-1999)			
Eotaxin (CCL11)				
Current:	87(55-128)	**0.001**	78(55-121)	0.013
Former:	59(34-90)		60(38-90)	
Eotaxin-2 (CCL24)				
Current:			623(240-786)	0.039
Former:			311(74-923)	
Gro-a (CXCL1)				
Current:	365(311-588)	**0.001**	365(319-587)	0.010
Former:	290(153-6)		298(153-497)	
CCL1/I-309				
Current:	93(75-283)	0.005		
Former:	73(75-283)			
IFNγ				
Current:	71(41-179)	0.034		
Former:	51(22-105)			
IL-1b				
Current:	8(5-34)	0.011		
Former:	7(2-12)			

IL-2				
Current:	27(16-76.6)	0.017		
Former:	19(7-41)			
IL-4				
Current:	31(23-91)	< **0.0001**	31(23-38)	0.013
Former:	21(13-38)		23(15-38)	
SDF-1a+b (CXCL12)				
Current:	2741(1713-4158)	**0.003**	2741(1713-4158)	0.010
Former:	1994(746-3212)		2290(1018-3212)	
TNFα				
Current:	60(37-125)	0.037		
Former:	45(5-147)			
PDGF-BB				
Current:	4127(2687-6244)	0.006	4127(2687-6344)	0.011
Former:	2949(461-6538)		2808(461-5444)	
VEGF				
Current:	125(74-425)	0.042		
Former:	92(6-304)			
MMP1				
Current:	2596(423-9385)	0.042	3022(10-9385)	0.026
Former:	1291(81-10606)		1291(319-6224)	
**Histology:**				
CXCL13				
Adenocarcinoma:	51(27-486)	0.029		
Squamous cell carcinoma:	72(30-842)			
Others:	356(54-414)			
MMP-12				
AC:			644(347-2982)	0.011
SCC:			1478(858-2752)	
Others:			No patients	
**Stage:**				
CXCL13				
III:			59(47-842)	0.015
IV:			48(27-187)	
CXCL1/ Fractalkine (CX3CL1)				
III:			349(165-490)	0.030
IV:			243(96-372)	
Gro-b (CXCL2)				
III:	675(155-947)	0.002	703(155-947)	0.010
IV:	1054(385-1641)		1029(385-1641)	
IP-10 (CXCL10)				
III:			388(157-520)	0.023
IV:			218(120-873)	
MIG (CXCL9)				
III:			1112(696-3578)	0.008
IV:			637(120-1390)	
IL-1ra				
III:	1050(14-1372)	0.039		
IV:	279(14-4162)			

TRAIL				
III:	69(22-641)	0.034		
IV:	51(16-164)			
MMP2				
III:	44002(27391-85148)	0.019		
IV:	34847(10902-66453)			
MMP-12				
III:			1911(644-3088)	0.026
IV:			904(37-2752)	
MMP-13				
III:	194(93-532)	0.006		
IV:	86(30-433)			
**ECOG:**				
TRAIL				
0:	79(63-98)	0.019		
1:	61(179-641)			
2:	42(16-113)			
**CRP:**				
CXCL2/Gro-a				
≤ 10	278(168-378)	0.031		
> 10	339(153-623)			
CXCL2/Gro-b				
≤ 10	592(208-1156)	**0.001**		
> 10	1029(155-1641)			
IL-4				
≤ 10	20(13-36)	0.029		
> 10	28(16-38)			
IL-6				
≤ 10	14(6-49)	< **0.001**	21(10-49)	0.023
> 10	29(13-103)		30(13-103)	
IL-8				
≤ 10	25(9-51)	0.008		
> 10	35(18-62)			
MDC (CCL22)				
≤ 10			1002(727-2566)	0.046
> 10			777(29-1813)	
MIP-1A (CCL3)				
≤ 10	13(8-23)	0.019		
> 10	16(9-29)			
IL-12p70				
≤ 10	49(6-160)	0.048		
> 10	45(16-226)			
PDGF-BB				
≤ 10	2628(1221-4256)	0.048		
> 10	3525(461-6538)			
VEGF				
≤ 10	68(6-199)	0.005		
> 10	138(20-425)			
MMP-1				
≤ 10	1009(375-4083)	0.009		
> 10	2095(81-10606)			
MMP-8				
≤ 10			4942(2883-9436)	0.004

> 10			10731(1337-121996)	
**Previous chemotherapy**				
MIP-1sigma (CCL15)				
Yes	10768(675-20703)	0.032		
No	8817(675-17060)			
MDC (CCL22)				
Yes	1058(183-1813)	0.010	1102 (553-1813)	0.006
No	646(179-2566)		748(299-2566)	
Fractalkine (CX3CL1)				
Yes	285(170-1250)	0.047	270(170-490)	0.022
No	234(96-530)		234(96-418)	
MMP-7				
Yes	4167 (1780-12000)	0.010	5396(1780-12000)	0.003
No	2678(755-6244)		2815(755-6244)	
MMP-8				
Yes	6899(1337-21857)	0.050		
No	12707(1569-121996)			
MMP-9				
Yes	28759(1289-115763)	0.050	26130(3826-59561)	0.045
No	56336(12823-152502)		49789(12823-113502)	

Concentration values measured in pg/ml. Abbreviations: NSCLC, non-small cell lung carcinoma; AC, adenocarcinoma; SCC, squamous cell carcinoma.

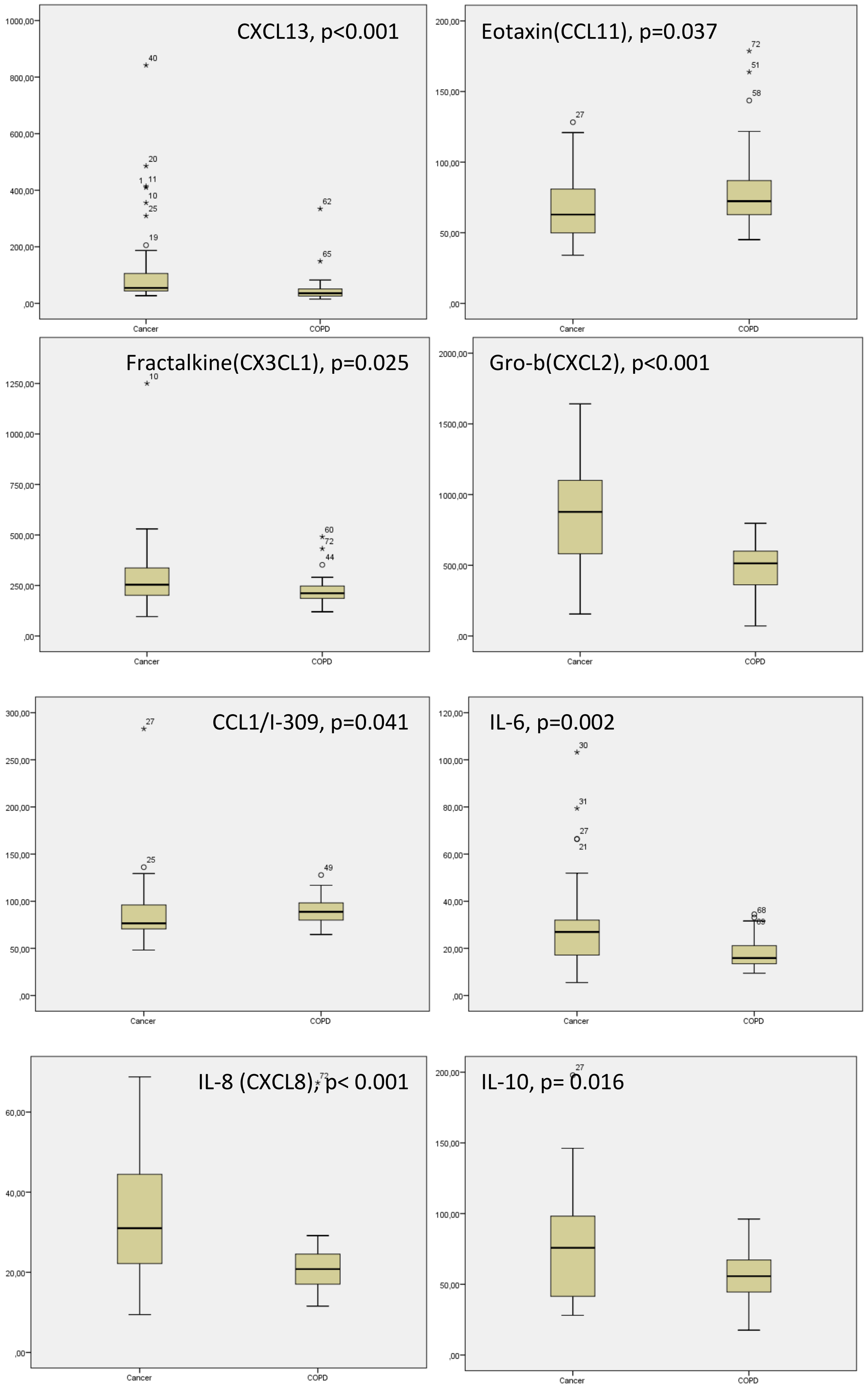

CXCL13, p<0.001
1000,00
800,00
600,00
400,00
200,00
,00
40
20
1
11
10
25
19
62
65
Cancer
COPD
Eotaxin(CCL11), p=0.037
200,00
150,00
100,00
50,00
,00
27
72
51
58
Cancer
COPD
Fractalkine(CX3CL1), p=0.025
1250,00
1000,00
750,00
500,00
250,00
,00
10
60
72
44
Cancer
COPD
Gro-b(CXCL2), p<0.001
2000,00
1500,00
1000,00
500,00
,00
Cancer
COPD
CCL1/I-309, p=0.041
300,00
250,00
200,00
150,00
100,00
50,00
,00
27
25
49
Cancer
COPD
IL-6, p=0.002
120,00
100,00
80,00
60,00
40,00
20,00
,00
30
31
27
21
68
Cancer
COPD
IL-8 (CXCL8), p< 0.001
60,00
40,00
20,00
,00
72
Cancer
COPD
IL-10, p= 0.016
200,00
150,00
100,00
50,00
,00
27
Cancer
COPD

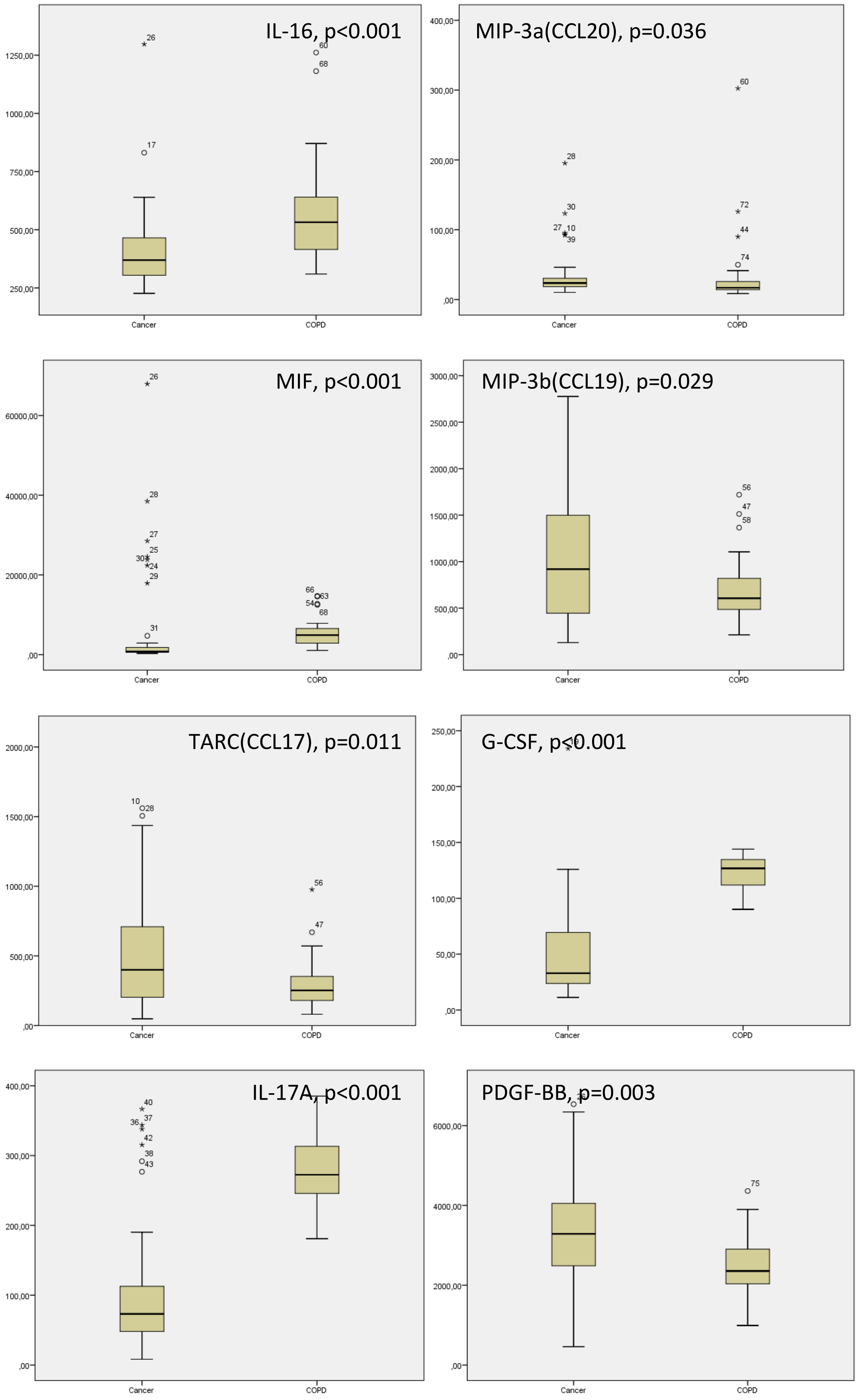
IL-16, p<0.001
1250,00
1000,00
750,00
500,00
250,00
Cancer
COPD
MIP-3a(CCL20), p=0.036
400,00
300,00
200,00
100,00
,00
Cancer
COPD
MIF, p<0.001
60000,00
40000,00
20000,00
,00
Cancer
COPD
MIP-3b(CCL19), p=0.029
3000,00
2500,00
2000,00
1500,00
1000,00
500,00
,00
Cancer
COPD
TARC(CCL17), p=0.011
2000,00
1500,00
1000,00
500,00
,00
Cancer
COPD
G-CSF, p<0.001
250,00
200,00
150,00
100,00
50,00
,00
Cancer
COPD
IL-17A, p<0.001
400,00
300,00
200,00
100,00
,00
Cancer
COPD
PDGF-BB, p=0.003
6000,00
4000,00
2000,00
,00
Cancer
COPD

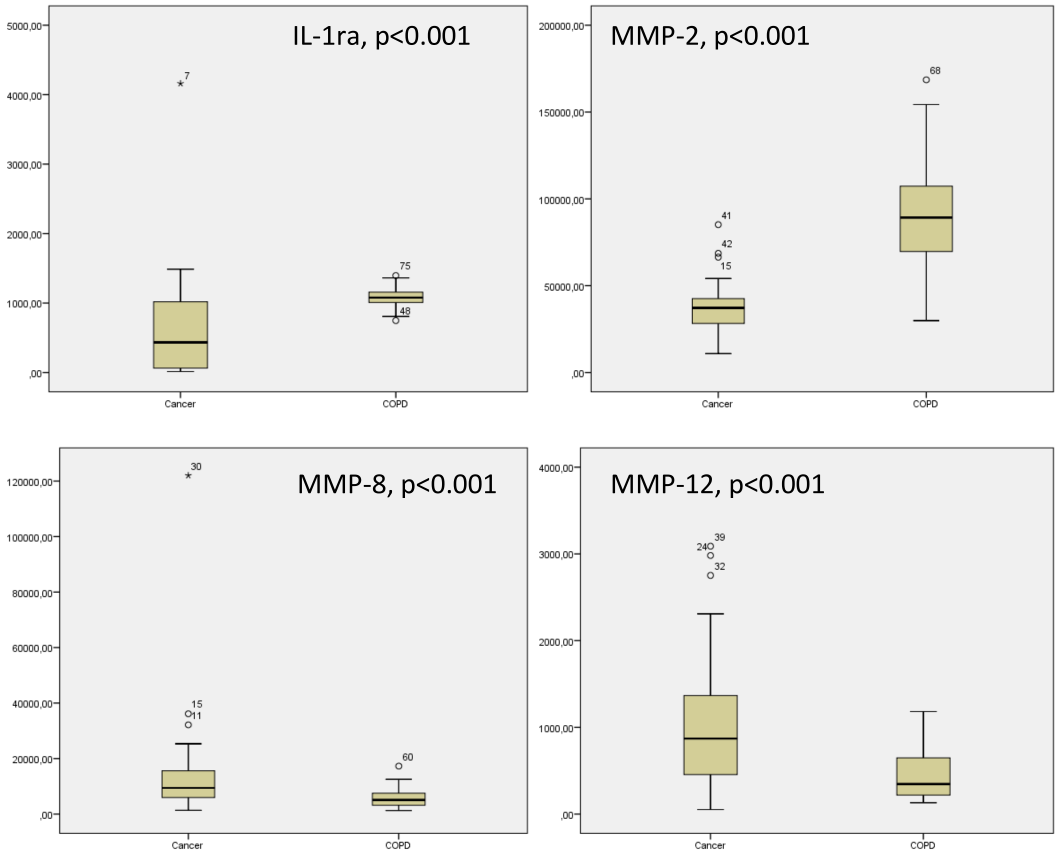

Figure S1. Box plots illustrating significant differences in cytokine concentrations in NSCLC and COPD patients. All concentration values (y-axis) measured in pg/ml.
